# Effects of SHBG rs1799941 Polymorphism on Free Testosterone Levels and Hypogonadism Risk in Young Non-Diabetic Obese Males

**DOI:** 10.3390/jcm8081136

**Published:** 2019-07-31

**Authors:** Daniel Castellano-Castillo, José Luis Royo, Ana Martínez-Escribano, Lidia Sánchez-Alcoholado, María Molina-Vega, María Isabel Queipo-Ortuño, Maximiliano Ruiz-Galdon, Juan J. Álvarez-Millán, Pablo Cabezas-Sanchez, Armando Reyes-Engel, Francisco J. Tinahones, Fernando Cardona, José C. Fernandez-Garcia

**Affiliations:** 1 Unidad de Gestión Clínica de Endocrinología y Nutrición del Hospital Virgen de la Victoria, Instituto de Investigación Biomédica de Málaga (IBIMA), Universidad de Málaga, 29010 Málaga, Spain; 2 Centro de Investigación Biomédica en Red de Fisiopatología de la Obesidad y la Nutrición, CIBERobn, 28029 Madrid, Spain; 3 Departamento de Especialidades Quirúrgicas, Bioquímica e Inmunología, Universidad de Málaga, 29010 Málaga, Spain; 4 Unidad de Gestión Clínica Intercentro de Oncología Médica del Hospital Virgen de la Victoria, Instituto de Investigación Biomédica de Málaga (IBIMA), Universidad de Málaga, 29010 Málaga, Spain; 5 Consulting Químico Sanitario SL (CQS Lab), 28521 Madrid, Spain

**Keywords:** SHBG, polymorphism, rs1799941, hypogonadism, obesity, free testosterone

## Abstract

Introduction: Obesity has been associated with increased risk of presenting hypogonadism. Free testosterone (FT) is the fraction of testosterone that carries out the biological function of testosterone, and is determined from total testosterone (TT) and sex-hormone binding globulin (SHBG) levels. We aimed to study the SHBG polymorphism rs1799941 in a cohort of young non-diabetic obese males to unravel the possible implication of this polymorphism in obesity-related hypogonadism. Methodology: 212 young (<45 years) non-diabetic obese (BMI ≥ 30 kg/m^2^) males participated in this study. Subjects were classified according to TT and FT levels in: Eugonadal (*n =* 55, TT > 3.5 ng/mL and FT ≥ 70 pg/mL; EuG), normal FT hypogonadism (*n =* 40, TT < 3.5 and FT ≥ 70 pg/mL; normal FT HG) and hypogonadism (*n =* 117, TT < 3.5 ng/mL and TL < 70 pg/mL; HG). The SHBG rs1799941 polymorphism (GG/GA/AA) was analyzed using the Taqman Open Array (Applied biosystem). Results: The rs1799941 frequencies were different among the groups. Higher proportion of the allele (A) was found in HG, compared to EuG and normal FT HG. Among the genotypes, the rare homozygous (AA) were found in the normal FT HG group and higher levels of serum SHBG and lower of FT were observed. The presence of the allele A was related (according to lineal regression models) to an increased of SHBG levels ((GA) β = 3.28; (AA) β = 12.45) and a decreased of FT levels ((GA) β = −9.19; (AA) β = −18.52). The presence of the allele (A) increased the risk of presenting HG compared to normal FT HG (OR = 2.54). Conclusions: The rs1799941 of the SHBG gene can partially determine the presence of obesity-related hypogonadism in young non-diabetic males and whether these subjects have normal FT HG.

## 1. Introduction

Obesity is considered one of the greatest health problems in developed countries. Closely associated with lifestyles, food intake and sedentary habits, obesity increases the risk of developing a wide range of comorbidities as type 2 diabetes (T2D), cardiovascular disease (CVD), metabolic syndrome and some type of cancers [1].

Obesity is also associated to an elevated prevalence of hypogonadism, comorbidity characterized by low levels of total testosterone (TT) or free testosterone (FT). Accordingly, in a cross-sectional based study carried out in 1849 US American men aged ≥45 years, more than 40% of non-diabetic obese men had low FT concentrations. In addition, the European Male Ageing Study (EMAS; carried out in 3369 men aged between 40–79 years) revealed that obese men had a decrease of up to 30% for TT levels and of up to 18% for FT levels compared with lean men [2].

Importantly, obesity-associated hypogonadism has been strongly associated with visceral adiposity, lean mass loss, metabolic syndrome, insulin resistance, T2D and cardiovascular disease [3,4].

Sex-hormone binding globulin (SHBG) is a protein synthesized in the liver and secreted into the blood stream where fulfills its biological function, which is the transport of sex steroids hormones. Therefore, SHBG levels emerge as one of the most critical parameters that are implied in regulating the access of these hormones to their target tissues, where it can even regulate testosterone action [5,6]. 

Most of the testosterone in plasma is usually bound to SHBG and albumin [7]. Nevertheless, only a fraction of the testosterone remains soluble in the plasma, and is accessible to the tissues, which is known as FT. Thus, plasma levels of FT are determined by the levels of TT and SHBG [7]. Indeed, low levels of TT with low levels of SHBG may result in normal levels of FT, whereas normal levels of TT with concomitant elevated SHBG levels may result in subnormal FT levels.

It has been described that serum levels of SHBG can be influenced by hormonal as well as nutritional and metabolic status [8]. Furthermore, it has been demonstrated that both serum levels of SHBG and TT are also determined by genetic factors [9,10,11,12]. Though, a genome-wide association study (GWAS) has demonstrated the relationship between several loci not only related directly to the SHBG gene but also to SHBG levels [13]. Recently it has also been shown that rare genetic variants for genes related to the pituitary–gonadotropic–gonadal axis function could play a role in the establishment of isolated hypogonadotropic hypogonadism [14]. Besides, the single nucleotide polymorphism (SNP) rs1799941 in the SHBG has been related to TT levels, with significantly lower TT levels and higher serum SHBG levels in men with the rare homozygous genotype [9]. The SNP rs1799941 is located in the SHBG promoter, just eight nucleotides before the transcriptional start site (TSS), and has been observed to affect SHBG gene expression and, in turn, SHBG plasma levels in several studies [9,12,15,16,17,18].

Moreover, in the last years FT levels assessment is gaining importance in the clinic practice due to the fact that subnormal FT levels have been related to a higher risk of Alzheimer disease, cardiovascular disease severity, endothelial function, atherosclerosis and a higher incidence of T2D [4,7,19,20,21]. Despite its importance, the knowledge about factors that could be involved in subnormal testosterone levels and SHBG levels in plasma are poorly understood. However, no study has yet been performed to analyze the possible relationship existing between the rs1799941 polymorphism and hypogonadism.

Therefore, in this study we aimed to examine the relationship between the genetic polymorphism rs1799941 in the SHBG gene and plasma concentrations of SHBG and testosterone among young non-diabetic obese males with different gonadal status.

## 2. Patients and Methods

### 2.1. Patients

From June 2013 to June 2015, primary care practitioners from six primary care centers in Malaga (Spain) consecutively invited young (<45 years) adult obese (defined by a body mass index (BMI, the weight in kilograms divided by the square of the height in meters) ≥30 kg/m^2^) males to participate in this study. 

Exclusion criteria for the present study were previous diagnoses of hypogonadism, diabetes mellitus, use of antidiabetic medication or being under any treatment known to affect the gonadal axis. Subjects with hepatic or renal impairment, CVD or cancer were also excluded. All subjects had a normal pubertal development and referred intact sense of smell.

In this study, participants were included and classified according to TT and FT levels: Eugonadal (EuG; TT ≥ 3.5 ng/mL), normal FT hypogonadism (normal FT HG; TT < 3.5 ng/mL, but FT ≥ 70 pg/mL) and hypogonadism (HG; TT < 3.5 ng/mL and FT < 70 pg/mL).

This study was reviewed and approved by the Ethics Committee of the Virgen de la Victoria University Hospital, and was conducted according to the principles of the Declaration of Helsinki. The participants (who were all volunteers) provided signed consent after being fully informed of the study goal and its characteristics.

### 2.2. Study Protocol

Study participants were instructed to eat a light meal the evening before the clinical evaluation and to fast with effect from 10 pm. Participants completed a structured interview to obtain demographic and clinical data, including height, waist circumference (WC) and blood pressure (BP). Blood samples were collected before 10 am and were centrifuged at 3130 g for 15 min at 4 °C. Plasma and serum were distributed in aliquots and stored at 80 °C until analysis.

Biochemical parameters were measured in duplicate by standard enzymatic methods. TT was determined by mass spectrometry (MS) coupled with high-performance liquid chromatography (HPLC; Triple Quadrupole LC/MS System–model 6460-, Agilent). The lower limit of detection was 0.024 ng/mL, the inter-assay coefficient of variation (CV) was 8.43% at 0.202 ng/mL, 2.64% at 1.49 ng/mL and 2.64% at 8.08 ng/mL and the intra-assay CV was 2.09% at 0.202 ng/mL, 3.67% at 1.49 ng/mL and 1.64% at 8.08 ng/mL. Accuracy was 103.7% and recovery was 97%. Sex hormone-binding globulin (SHBG) determination was done with an electrochemiluminescence immunoassay (Elecsys SHBG, Roche). Lower detection limit = 0.350 nmol/L and intra and inter-assay CV was 2.9% and 3.3% respectively. Reference ranges for TT in males were 3.5–14.5 ng/mL and 15–50 nmol/L for SHBG. Calculated FT was estimated from TT and SHBG by using Vermuelen’s formula [22]. In agreement with previous studies, we considered subnormal FT levels values lower than 70 pg/mL [23,24]. Luteinizing hormone (LH) was determined by a direct quimiluminometric assay (ADVIA Centaur, Siemens) and reference values for males were 1.5–9.3 mIU/mL. Insulin was analyzed by an immunoradiometric assay (BioSource International, Camarillo, CA) in a Beckman Coulter (Fullerton, CA), showing 0.3% cross-reaction with proinsulin. We used the homeostasis model assessment insulin resistance index (HOMA-IR), in order to determine the status of insulin resistance [25]. 

#### Polymorphism DNA Analysis

Genotyping was outsourced to Genologica SL. SNP analysis was performed using the TaqMan Open Array Genotyping System from Applied Biosystems as previously described. The results obtained were processed using TaqMan Genotyper Software. The selected SNP, those affecting the SHBG activity, was chosen from the literature [9,12,15,16,17,18].

### 2.3. Statistical Analyses

Statistical analysis was performed using the SPSS/PC statistical package (version 15 for Windows; SPSS, Chicago, IL, USA). Normal distribution of the variables was evaluated using the Kolmogorov–Smirnov test. For clinical and anthropometric variables, normal distributed data was expressed as mean value ± SD. Differences among groups were tested using ANOVA analyses follow by Duncan’s post-hoc analyses for parametric variables (total cholesterol, LDL cholesterol and hematocrit) while for the rest (non-parametric variables) Kruskal–Wallis followed by a Mann–Whitney U test was performed. Associations between the qualitative characteristics were tested by a chi-squared test. Lineal regression analyses were performed to study the association between FT and serum SHBG levels to the SNP for which dummy variables were performed to introduce the genotype as independent variables. The GG genotype was established as a reference genotype to calculate the weight of the genotype over SHBG and FT plasma levels. Logistic regression models were used to estimate the odds ratios (ORs) of presenting HG compared to EuG and normal FT HG. In order to calculate the influence of the genotype to explain these ORs, a dichotomist variable was created, modeling a dominant effect (presence or absence of allele A). Variables included in the logistic regression model were those that were statistically significant according to univariate analyses or were biologically relevant. All *p* values were based on a two-sided test of statistical significance. Significance was accepted at the level of *p* ≤ 0.05.

## 3. Results

In this study 212 subjects were included; 110 patients were included in the EuG group, 60 in the normal FT HG group and 51 in the HG group. Anthropometric and biochemical variables between study groups are shown in Table 1. Differences among the three groups were found for BMI, waist circumference, TT and FT. We also observed higher insulin, HOMA-IR, CRP and HbA1c levels in HG and normal FT HG with respect to the EuG group, while LH levels were different between the EuG and the HG groups. 

After analyzing the frequency distribution for the alleles of the SNP rs1799941 we tested that they were in Hardy Weinberg equilibrium, being 0.91 the frequency for the major allele (G) and 0.48 for the minor allele (A). The genotypic frequency distribution is shown in Table 2. Thus, we observed a higher percentage of the rare allele (A) in HG subjects with respect to EuG subjects and normal FT HG subjects specially. Furthermore, no homozygotes subjects for the rare allele (AA) were found in the normal FT HG group.

**Figure 1:**
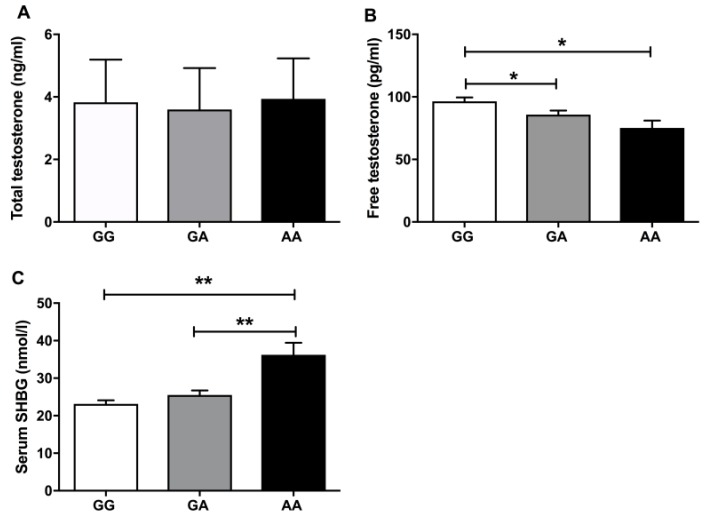
(**A**) The mean of the total testosterone levels across each SHBG polymorphism. (**B**,**C**) The mean distribution of free testosterone and serum SHBG for each and every study genotype. * means *p* < 0.05 and ** means *p* < 0.01 according to Kruskal–Wallis statistics followed by a Mann–Whitney U test for the group by group comparisons.

Next, we evaluated TT and FT serum levels among the studied genotypes. We did not found significant differences among the three genotypes (GG, GA and AA) for TT levels (Figure 1A), but there were lower levels of FT in the rare genotype AA and GA compared to the wild type GG (Figure 1B). According to serum SHBG levels, there was an increase of the globulin in the homozygous genotype AA, in comparison with the genotypes GG and GA (Figure 1C).

This relationship between FT and the rs1799941 genotype was also evaluated through a lineal regression analysis. Thus, in a lineal model adjusted by age, BMI, HOMA-IR and LH, the presence of the rare allele A was associated with a significant decrease in FT, that was even more pronounced when the homozygote genotype (AA) was found (Table 3). Moreover, a lineal regression model with serum SHBG levels as dependent variable and corrected by age, BMI, HOMA-IR, LH and TT, the rs1799941 polymorphism was able to explain the serum expression of this globulin. Concretely, both the presence of the heterozygous genotype (GA) and the presence of the rare genotype (AA) were associated with a significant increase in serum SHBG levels (Table 4).

Lastly, we also analyzed the role of the polymorphism rs1799941 in the potential diagnosis of obesity-related hypogonadism. In this regard, a logistic regression analysis showed that the presence of the rare allele (A) was associated with a 2.5-fold in the OR of having hypogonadism, in comparison with having normal FT HG (Table 5), while no significant association according to the genotype was found either when EuG/Normal FT HG nor EuG/HG were compared. 

## 4. Discussion

**Figure 2:**
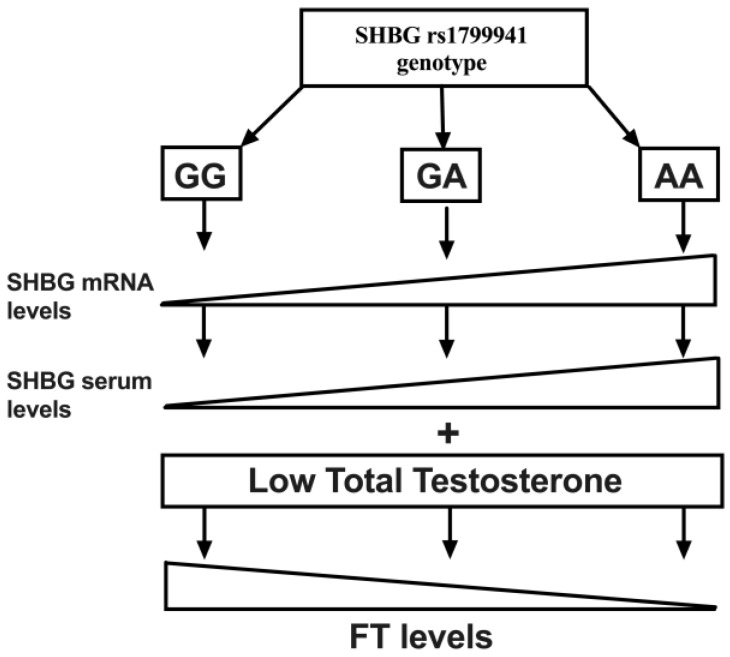
Schematic representation showing the relationship between the genotype of the rs1799941 and the gene and serum expression of SHBG. The presence of the minor allele (A) triggers a higher expression of both mRNA and serum levels of SHBG, which at the same levels of total testosterone (TT) can reduce free testosterone (FT) levels. Thus, the presence of the minor genotype (AA) could hinder FT compensation capacity at a low TT level state.

In this study we described for the first time a relationship between the SHGB polymorphism rs1799941 not only with SHBG serum levels, but also with FT levels. We also described the frequency of this polymorphism in a young non-diabetic obese population. In this line, although we observed the presence of the minor allele (A) in the three studied groups, significant differences in the genotype distribution were found. Accordingly, in our study the presence of the minor genotype (AA) was associated with reduced FT levels, despite similar TT concentrations (Figure 2). 

Serum levels of SHBG are influenced by hormonal, nutritional and metabolic status [8]. Besides, the genetic background could also be responsible in part of SHBG levels. In this line, the SHBG SNP rs1799941 A/G has been shown to be a genetic determinant for serum SHBG levels [12,15,18]. This SNP might have a direct impact on SHBG transcription since it is located within the human SHBG proximal promoter sequence (eight base pairs from the transcription start site), which would agree with the phenotype observed in previous studies [12,15,18]. In this sense, in our study we have described a significant increase in the serum SHBG levels in the presence of the minor genotype (AA).

On the other hand, total blood stream testosterone is mostly bound to SHBG and in a less extent to albumin, while only a fraction between 2%–5% remains free. This free fraction is supposed to be able to enter the cell in order to carry out its biological function [26]. Since rs1799941 A/G affects the serum levels of SHBG, a relationship between the genotype and serum FT levels should be expected. However, none of the studies where this rs1799941 variant has been studied have found a relationship between FT and the genotype, which could be due to the fact that these studies have been performed in heterogeneous study populations. By contrast, we have analyzed the possible effect of this SNP over serum SHBG and FT in a homogenous sample population, with no interferences concerning ageing, or the presence of T2D or cardiovascular disease, comorbidities frequently associated in the obese male.

Our results show that 24.5% of all the study population had levels of FT under 70 pg/mL. Moreover, we found an association between the SHBG polymorphism rs1799941 and FT levels, being the FT levels lower in those subjects with the rare genotype (AA) respect to the heterozygous (GA) and the major homozygous (GG) genotypes (Figure 2). This association was maintained in the regression analysis, even though this analysis was corrected with variables strongly associated to FT as age, BMI, HOMA-IR and LH. The SHBG polymorphism turned out to be the best variable that could explain these FT levels. Thus, we proposed that this relationship between the SHBG genotype and FT levels in young non-diabetic obese people might be due to the influence of the genotype over serum SHBG levels. Altogether this data could explain in part the hypogonadism associated with obesity and the absence of this genotype (AA) in the normal FT HG group.

In our study, serum SHBG was predicted by age, TT and the rs1799941 SHBG polymorphism, but no associations were found with BMI and HOMA-IR, possibly due to our study population, where only young males without T2D were included. For instance, it has been described that a decrease of TT was associated with age, while the inverse tendency was observed in serum SHBG levels. This leads as a result, to a lowering of FT and consequently to a loss of testosterone-associated activity with aging [27,28,29].

TT has been shown to decrease with obesity together with SHBG levels, producing compensation and no changes in FT [24,30]. Due to this relationship between SHBG and TT levels with obesity, FT levels, which have been related to hypogonadism symptoms [31,32], would be a suitable variable to diagnose the hypogonadism in obese subjects [33]. Accordingly, as we show in the logistical regression model, even though BMI could affect to the compensation observed in the groups of subnormal TT levels (normal FT HG and HG), the presence or absence of the allele A emerged as a strong variable that could explain this compensatory mechanism. 

In conclusion, in this study we found that the rs1799941 polymorphism in young non-diabetic obese males with hypogonadism was related to SHBG levels and consequently could be determining the FT fraction according to the relationship present between FT and the rs1799941 polymorphism as well. Moreover, in this study we show that the SHBG rs1799941 polymorphism differed among types of obesity-related hypogonadism in young non-diabetic males and might provide discriminatory potential to identify subjects with normal FT HG. Thus, our study described a genetic factor that could be of clinical interest in the management of obesity-related hypogonadism.

## Figures and Tables

**Table 1:** Anthropometric and analytical variables among the study groups. Values are given as the means and SD.

	Eugonadal (*n =* 101)	Normal FT HG (*n =* 60)	HG (*n =* 51)
Age (years)	37.62 ± 7.65	36.12 ± 7.35	38.41 ± 7.59
Smokers (%) *	31	19	15
BMI (kg/m^2^)	36.76 ± 5.29**a**	38.63 ± 5.76**b**	44.49 ± 8.42**c**
Waist (cm)	119.55 ± 13.11**a**	123.84 ± 13.33**b**	136.11 ± 17.83**c**
Glucose (mg/dl)	91.39 ± 9.95	93.12 ± 11.33	93.25 ± 10.38
Insulin (μU/mL)	16.36 ± 8.05**a**	22.45 ± 11.65**b**	25.58 ± 18.70**b**
HOMA-IR	3.74 ± 2.04**a**	5.41 ± 6.48**b**	5.95 ± 4.40**b**
Triglycerides (mg/dl)	151.26 ± 81.68	165.12 ± 72.19	151.12 ± 81.93
Chol (mg/dl)	191.75 ± 34.68	185.73 ± 32.83	179.75 ± 29.46
HDL (mg/dl)	42.94 ± 10.47	39.93 ± 7.04	41.02 ± 9.59
LDL (mg/dl)	119.70 ± 29.57	114.20 ± 28.35	110.10 ± 24.81
CRP (mg/L)	5.12 ± 3.63**a**	6.74 ± 5.77**b**	8.50 ± 6.09**b**
HbA1c (%)	5.32 ± 0.36**a**	5.46 ± 0.32**b**	5.52 ± 0.35**b**
Hematocrit (%)	46.50 ± 2.74	45.66 ± 3.02	45.65 ± 3.09
TSH (μU/mL)	1.79 ± 0.97	1.81 ± 0.81	1.89 ± 0.93
FSH (mUI/mL)	4.18 ± 2.43	3.89 ± 2.31	3.52 ± 2.09
LH (mUI/mL)	4.10 ± 1.62**a**	3.79 ± 1.62**a,b**	3.21 ± 1.62**b**
Estradiol (pg/mL)	33.77 ± 12.58	31.56 ± 14.76	34.10 ± 13.39
Testosterone (ng/mL)	4.83 ± 1.10**a**	3.04 ± 0.32**b**	2.41 ± 0.54**c**
FT (pg/mL)	111.33 ± 30.99**a**	84.22 ± 9.54**b**	56.90 ± 9.85**c**
SHBG (nmol/L)	30.00 ± 11.60**a**	17.48 ± 5.47**b**	24.82 ± 10.30**c**

Eugonadal subjects (EuG); normal FT hypogonadism subjects (normal FT HG); hypogonadism subjects (HG); body mass index (BMI); homeostatic model assessment of insulin resistance (HOMA-IR); total cholesterol (Chol); high-density lipoprotein cholesterol (HDL); low-density lipoprotein (LDL), C-reactive protein (CRP); glycated hemoglobin (HbA1c); prostate-specific antigen (PSA); thyroid-stimulating hormone (TSH); follicle-stimulating hormone (FSH); luteinizing hormone (LH); free testosterone (FT); sex hormone binding globulin (SHBG). * means *p* < 0.05 according to a chi squared test for frequencies. Different letters means significant differences (*p* < 0.05) according to an ANOVA followed by Duncan’s test post-hoc for normal variables or Kruskal–Wallis followed by a Mann–Whitney U test for non-normal variables.

**Table 2:** Genotype for the SHBG polymorphism rs1799941 among the different groups of study. Values are given as the percentage of the frequencies.

	SHBG rs1799941 Polymorphism (%)		
	*GG*	*GA*	*AA*
Eugonadal	52.5	36.6	10.9
Normal FT HG	58.3	41.7	0.0
HG	41.2	43.1	15.7

Sex-hormone binding protein (SHBG). Eugonadal group; normal FT hypogonadism group (normal FT HG); hypogonadism group (HG). Chi squared test: *p* = 0.032.

**Table 3:** Lineal regression analysis with FT as dependent variable, and age, BMI, HOMA-IR, LH and the polymorphism variants of the SHBG rs1799941 as independent parameters.

	Free Testosterone (*R* = 0.442. *R*^2^ = 0.195)		
	β	*p*	95% CI
Age (years)	−0.440	0.099	−0.964–0.083
BMI (kg/m^2^)	−1.448	0.000	−2.057–(−0.839)
HOMA-IR	−0.186	0.704	−1.146–0.775
LH (mUI/mL)	3.305	0.007	0.925–5.685
SHBG rs1799941_GA	−9.950	0.020	−18.335–(−1.564)
SHBG rs1799941_AA	−17.994	0.016	−32.664–(−3.324)

Body mass index (BMI); homeostatic model assessment of insulin resistance (HOMA-IR); luteinizing hormone (LH); sex hormone binding globulin (SHBG).

**Table 4:** Lineal regression analysis with serum SHBG levels as dependent variable, and age, BMI, HOMA-IR, LH, testosterone and the polymorphism variants of the SHBG rs1799941 as independent parameters.

	Serum SHBG (*R* = 0.640. *R*^2^ = 0.410)		
	β	*p*	95% CI
Age (years)	0.391	0.000	0.232–0.551
BMI (kg/m^2^)	0.155	0.121	−0.041–0.351
HOMA-IR	−0.46	0.326	−0.440–0.147
LH (mUI/mL)	−0.032	0.931	−0.771–0.706
Testosterone (ng/mL)	4.128	0.000	3.147–5.110
SHBG rs1799941_GA	3.103	0.018	0.542–5.664
SHBG rs1799941_AA	11.695	0.000	7.230–16.161

Body mass index (BMI); homeostatic model assessment of insulin resistance (HOMA-IR); luteinizing hormone (LH); sex hormone binding globulin (SHBG).

**Table 5:** Logistic regression analysis adjusted by age, BMI and HOMA-IR. Likelihood of presenting hypogonadism.

	Normal FT HG/HG *R*^2^ = 0.197–0.264	
	OR (95% CI)	*p*
Age	1.05 (0.99–1.11)	0.082
BMI	1.13 (1.06–1.21)	0.000
HOMA-IR	0.95 (0.88–1.04)	0.306
SHBG rs1799941		
Absence A	1 (reference)	
Presence A	2.54 (1.05–6.12)	0.037

95% Confidence intervals (95% CI); body mass index (BMI); sex hormone binding globulin (SHBG); normal FT hypogonadism (normal FT HG); hypogonadism (HG).
